# *MDN1* variants cause susceptibility to epilepsy: For the China Epilepsy Gene 1.0 Project

**DOI:** 10.1186/s42494-025-00209-3

**Published:** 2025-03-03

**Authors:** Qianru Wen, Dongming Zhang, Yan Ding, Sheng Luo, Qiang Huang, Junhui Zhu, Yongxin Li, Wenhui Liu, Pengyu Wang, Xian Li, Zisheng Lin, Yaying Wang, Xiaoyu Liang, Weiping Liao, Jie Wang, Heng Meng

**Affiliations:** 1 Department of Neurology, The Sixth Affiliated Hospital of Jinan University, Dongguan, 523573 China; 2 Department of Neurology, Institute of Neuroscience, Key Laboratory of Neurogenetics and Channelopathies of Guangdong Province and the Ministry of Education of China, the Second Affiliated Hospital, Guangzhou Medical University, Guangzhou, 510260 China; 3 Department of Neurology, The First Affiliated Hospital of Jinan University, Guangzhou, 510632 China; 4 Department of Pediatric, Xiangxi Tujia and Miao Autonomous Prefecture People’s Hospital, Jishou, 416000 China; 5 Department of Neurology, The Seventh Affiliated Hospital of Sun Yat-Sen University, Shenzhen, 518000 China; 6 Department of Neurology, Shunde Hospital of Southern Medical University (the First Hospital of Shunde), Shunde, 528308 China; 7 Department of Pediatric, Zhuhai Women’S and Children’S Hospital, Zhuhai, 519000 China

**Keywords:** *MDN1* variants, Epilepsy, Susceptibility, Molecular subregional implication, Febrile seizures, Brain damage

## Abstract

**Background:**

The Midasin AAA (ATPase associated with various activities) ATPase 1 (*MDN1*) gene, a member of the AAA protein family, plays a crucial role in ribosome maturation. *MDN1* is expressed in the human brain throughout life, especially during early development and adulthood. However, *MDN1* variants have not been previously reported in patients with epilepsy. This study aims to explore the association between *MDN1* variants and epilepsy.

**Methods:**

Trios-based whole-exome sequencing was performed in a cohort of patients with epilepsy susceptibility from the China Epilepsy Gene 1.0 Project. The excess, damaging effects, and molecular subregional implications of variants, as well as the spatio-temporal expression of *MDN1*, were analyzed to validate the gene-disease association.

**Results:**

Compound heterozygous variants in *MDN1* were identified in five unrelated patients with febrile seizures or secondary epilepsy. Three patients presented with febrile seizures/epilepsy with febrile seizures plus, while two patients developed epilepsy secondary to brain damage (five or seven years after). These variants were either absent or present at low frequencies in the control group, and exhibited statistically significant higher frequencies in the case group compared to controls. All the missense variants were predicted to be damaging by at least one in silico tool. In each pair of compound heterozygous variants, one allele was located in the AAA2-AAA3 domains, while the other allele was located in the linker domain or its vicinity. In contrast, most of the variants from the asymptomatic control group were located outside the AAA domains, suggesting a molecular subregional implication of the *MDN1* variants.

**Conclusions:**

*MDN1* is potentially a susceptibility gene for epilepsy.

**Supplementary Information:**

The online version contains supplementary material available at 10.1186/s42494-025-00209-3.

## Background

Epilepsy is a common neurological disorder characterized by complex etiologies and diverse clinical manifestations. Genetic factors, brain structural abnormalities, perinatal factors, infections, and trauma are among the causes associated with epilepsy. Notably, epilepsy may be associated with monogenic abnormalities. Genetic factors account for up to 80% of idiopathic epilepsy cases and are considered primary causative factors [1–3]. In cases of epilepsy secondary to acquired brain damage, genetic factors may play a role in the alterations of susceptibility to epilepsy. In recent decades, substantial advancements have been achieved in the identification of genes responsible for epilepsy [4]. Genes associated with epilepsy are involved in ion channels, neurotransmitters, synapses, cellular cytoskeletons, as well as transcription regulation functions [5, 6]. However, genes related to epilepsy susceptibility are rarely reported. Although a few studies have reported associations between single nucleotide polymorphisms (SNPs) and epilepsy through large-scale genome-wide association studies (GWAS), such associations warrant validation in individuals with epilepsy.

The ribosome is an important organelle responsible for protein synthesis, influencing a series of activities such as growth and reproduction [7]. The Midasin AAA (ATPase associated with various activities) ATPase 1 (*MDN1*) gene (OMIM*618200), encoding an AAA ATPase, belongs to the AAA protein family. It is predicted to remove assembly factors from distinct precursors of the ribosomal 60S subunit and plays a key role in the process of ribosome maturation [8–10]. *MDN1* is expressed in the human brain throughout whole life, especially during early development and adulthood. However, the association between *MDN1* and human disease remains unclear.

In the present study, we conducted trio-based whole-exome sequencing (WES) in a group of patients with febrile seizures (FS) or epilepsy secondary to brain abnormalities from the China Epilepsy Gene 1.0 Project. Five pairs of compound heterozygous variants in *MDN1* were identified in five unrelated family cases, each involving one affected individual. This study suggests that *MDN1* is potentially a susceptibility gene for epilepsy.

## Methods

### Participants

Patients were recruited from the China Epilepsy Gene 1.0 Project (https://epg1.cn), which was divided into 16 groups. A total of 291 patients in the present study were from the groups of FS, epilepsy with febrile seizures plus (FS +) and susceptibility of epilepsy. For patients with FS + , only those presenting with afebrile generalized tonic–clonic seizures (GTCS) were included. For patients with epilepsy secondary to brain damage, epilepsy onset occurred at least one year after the initial diagnosis of the primary disease. Patients with other pathogenic or potentially pathogenic genetic variants related to epilepsy [4], such as *SCN1A* variants, were excluded. Detailed clinical data were collected for all affected patients, including comprehensive clinical phenotypes, gender, age of seizure onset, current age, seizure types and course, acquired causes, anti-seizure medications (ASMs), response to ASMs, growth and development evaluation, long-term video electroencephalogram (EEG) monitoring, magnetic resonance imaging (MRI), as well as any associated complications. Seizure types were categorized according to the principles established by the Commission on Classification and Terminology of the International League Against Epilepsy (ILAE) [11–16].

The project followed the guidelines set by the International Committee of Medical Journal Editors concerning patient consent for research participation. Ethical approval was obtained from the Ethics Committee of Guangzhou Medical University Second Affiliated Hospital (No. 2020-hs-49), and written informed consent was obtained from the legal guardians of the participants.

### Whole-exon sequencing and genetic analysis

To determine the origin of genetic variants, blood specimens were collected from the probands, as well as their respective fathers and mothers. Genomic DNA extraction was conducted using the Qiagen Flexi Gene DNA kit (Hilden, Germany). WES was performed on Illumina HiSeq 2000 platform (Illumina, San Diego, California, USA). The raw sequencing data were then accurately aligned to the Genome Reference Consortium Human Genome build 37 (GRCh37) using Burrows-Wheeler Aligner. Subsequently, the identification of single-nucleotide variants and indels was conducted with the Genome Analysis Toolkit. Detailed sequencing procedures have been described in previous studies [17–22].

In accordance with the methodology previously described, a case-by-case analytical approach was employed to detect potential disease-causing variants within each trio [23–26]. Initially, variants with a minor allele frequency (MAF) of more than 5 × 10^–3^ in the gnomAD database were excluded from subsequent evaluation. Potentially disease-causing variants were selected for further analysis, such as canonical splicing, nonsense, frameshift, in-frame indels, missense, and initiation codon variants [23, 24, 27]. The variants were further filtered based on their interpretable inheritance patterns within the trio, focusing on compound heterozygous, hemizygous, homozygous, or de novo variants. Subsequently, stratified criteria for MAF were applied: hemizygous, homozygous, and de novo variants must be absent in the control cohort from gnomAD. For compound heterozygous variants, the product of the frequencies of the biallelic in gnomAD must be below 1 × 10^–6^, a value considerably lower than the expected probability of such an occurrence in the current gnomAD population (1/141456 = 7 × 10^–6^). Ultimately, the candidate variants were refined using strict criteria from the GD&P Database. These criteria comprehensively encompassed four pivotal facets of the gene profiles [19]: 1) tissue-specific expression; 2) exclusion of conflicting gene-disease associations; 3) evaluation of gene intolerance to variants; and 4) phenotypic consequences of genetic manipulation.

Following the application of filtering criteria, recurrent variants were screened for further analysis to clarify the specific gene-disease associations [19, 28–31]. *MDN1* emerged as a potential candidate gene for these recurrent variants. All identified *MDN1* variants in this study were confirmed through Sanger sequencing and annotated to NM_014611.

### Excess analysis of *MDNI* variants

To explore the association between *MDN1* and susceptibility to epilepsy, specific statistical methods were employed, focusing on the frequency of compound heterozygous variants in the asymptomatic control group and the aggregate frequency of variants in the gnomAD database (gnomAD V2.1.1). A cohort of 1942 asymptomatic parents was utilized as the control group to assess the significance of biallelic variants in patients. In this cohort, compound heterozygous variants were detected by identifying one of the paired variants in the child, based on the fact that one variant from a parent would be inherited by the child. For aggregate frequency evaluation, the variant frequencies observed in the patient group were compared with the population frequencies in the gnomAD database.

### Damaging effect analysis

To evaluate the potential damaging effects of the candidate variants on the molecular structure, protein modelling was carried out utilizing the AlphaFold protein structure database. The three-dimensional protein structures were analyzed and visualized using the PyMOL Molecular Graphics System (Version 2.3.2, Schrödinger, LLC). The I-Mutant 2.0 program was employed to estimate the effect of *MDN1* missense variants on protein stability, quantifying the effects by measuring changes in free energy (DDG, kcal/mol). Values exceeding 0.5 kcal/mol indicate a significant enhancement in protein stability, while a value below − 0.5 kcal/mol suggests a substantial reduction in protein stability; values between these thresholds are considered neutral [32]. Nine in silico tools were applied to predict the damaging effects of all missense variants, including MutationTaster, CADD, fitCons, ReVe, GERP + + , phyloP, PhastCons, Fathmm-MKL, and SiPhy. The alterations in hydrophobicity among the missense variants were assessed using the VarSite web server [33].

### Temporal expression of *MDN1*

The expression profiles of *MDN1* and its developmental stages were studied using data from the Evo-devo mammalian organs database and the BrainSpan database. The expressional spline was fitted by the locally weighted scatterplot smoothing (LOWESS) algorithm to interpret the expression pattern of *MDN1*.

### Statistical analysis

SPSS version 23 was used for data processing. The two-tailed Fisher's exact test was conducted to assess the comparison of variant frequencies between the case cohort and controls. A* P*-value threshold of less than 0.05 was considered statistically significant.

## Results

### Identification of *MDN1* variants

**Fig. 1:**
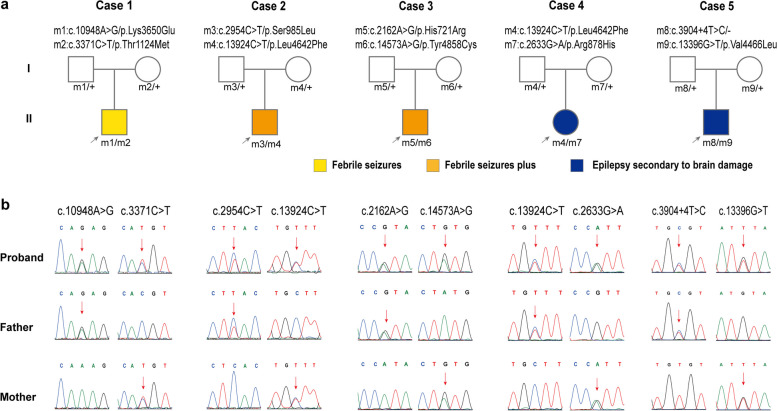
Genetic data of cases with *MDN1* variants. **a** Pedigrees of the five cases with *MDN1* variants and their corresponding phenotypes. **b** DNA sequence chromatograms of the *MDN1* variants. Arrows indicate the positions of the mutations

Five pairs of compound heterozygous variants in *MDN1* were identified in five family cases, including eight missense variants (c.2162A > G/p.His721Arg, c.2633G > A/p.Arg878His, c.2954C > T/p.Ser985Leu, c.3371C > T/p.Thr1124Met, c.10948A > G/p.Lys3650Glu, c.13396G > T/p.Val4466Leu, c.13924C > T/p.Leu4642Phe, c.14573A > G/p.Tyr4858Cys) and one splicing variant (c.3904 + 4 T > C) (Fig. 1 and Table 1). Among them, the missense variant c.13924C > T/p.Leu4642Phe was recurrently observed in Cases 2 and 4. All *MDN1* compound heterozygous variants were transmitted by their asymptomatic parents, consistent with the pattern of Mendelian autosomal recessive inheritance.

**Table 1:** Clinical features of the cases with *MDN1* variants

Cases	Variants (NM_014611)	Gender	Age	Onset Age	Seizure course	Acquired cause	ASMs	EEG	Brain MRI	DD	Seizure outcome	Diagnosis
Case 1	c.10948A > G/p.Lys3650Glu c.3371C > T/p.Thr1124Met	M	29y	1y	FS, once	Normal	-	NA	NA	Normal	Seizure free	FS
Case 2	c.2954C > T/p.Ser985Leu c.13924C > T/p.Leu4642Phe	M	9y	2y	FS, 3 times GTCS, once	Normal	VPA	Spikes, spike-slow waves in right parieto-occipital region	Normal	Normal	Seizure free	FS +
Case3	c.2162A > G/p.His721Arg c.14573A > G/p.Tyr4858Cys	M	7y	1y	FS, twice GTCS, once	Normal	VPA	Generalized sharp-slow waves	Normal	Normal	Seizure free	FS +
Case 4	c.13924C > T/p.Leu4642Phe c.2633G > A/p.Arg878His	F	10y	7y	SPS & sGCTS, 4–5 times/day	ABO hemolysis at birth	OXC, PER	Multifocal spikes, spike-slow waves	Bilateral parietal cortex atrophy and encephalomalacia	DD	Refractory	FE
Case 5	c.3904 + 4 T > C/- c.13396G > T/p.Val4466Leu	M	21y	6y	CPS, 3–4 times/day	Infection and SAH 9 months of age	NA	Multifocal sharp-slow and spike-slow waves	Left hemisphere atrophy	DD	Refractory	FE

*Abbreviations*: *ASMs* Anti-seizure medications, *CPS* Complex partial seizure, *DD* Developmental disorder, *EEG* Electroencephalogram, *FE* Focal epilepsy, *FS* Febrile seizures, *FS* + Febrile seizures plus, *GTCS* Generalized tonic–clonic seizure, *MRI* Magnetic resonance imaging, *NA* Not available, *OXC* Oxcarbazepine, *PER* Perampanel, *sGTCS* secondarily generalized tonic–clonic seizure, *SAH* Subarachnoid hemorrhage, *SPS* Simple partial seizure, *VPA* Valproate, *y* year, *m* month

The identified *MDN1* variants were either absent or present low frequencies (minor allele frequency < 5 × 10^–3^) in the gnomAD-all or gnomAD-East Asian populations. None of them were present in a homozygous state in the gnomAD-all population.

**Fig. 2:**
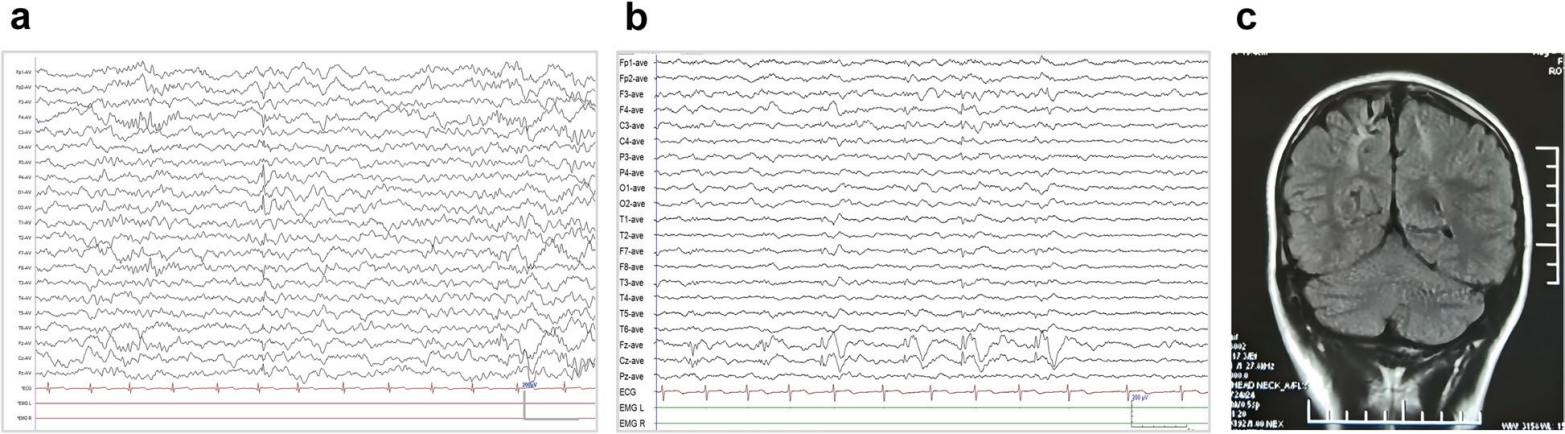
Representative EEGs and MRI of patients with *MDN1* variants. **a** Interictal EEG of Case 2 showed spike waves in the right parieto-occipital region at the age of 8 years. **b** Interictal EEG of Case 4 showed spikes and spike-slow waves in the bilateral frontal, central, and midline regions at the age of 9 years. **c** The MRI of Case 4 showed cortical atrophy and encephalomalacia in the bilateral frontal and parietal lobes

In the case cohort, the aggregate frequency of *MDN1* variants was discovered to be significantly greater than in the controls (Table 2), which included comparisons with the gnomAD-all population (10/582 vs. 189/250470, *P* < 0.001), the East Asian subgroup within the gnomAD (10/582 vs. 151/18324, *P* = 0.034), the control group from the gnomAD-all population (10/582 vs. 71/106914,* P* < 0.001), and the control group from the East Asian subgroup within the gnomAD (10/582 vs. 61/9036, *P* = 0.01) (Table 2). In the control cohort of 1942 asymptomatic parents, nine pairs of compound heterozygous variants in *MDN1* were identified (Fig. 2a). The frequency of variants in this study cohort was significantly greater compared to that in the control group. (5/291 vs. 9/1942; OR = 3.75, 95%CI 1.25–11.28; *P* = 0.027).

**Table 2:** Analysis of the aggregate frequency of *MDN1* variants identified in this study

Variants	Allele count/number in this study	Allele count/ gnomAD-all	Allele count/ gnomAD-control	Allele count/ gnomADEas-all	Allele count/ gnomADEas-control	Homozygotes/gnomAD-control
c.2162A > G/p.His721Arg	1/582	6/282232 (0.00002126)	3/109224 (0.00002747)	5/19926 (0.0002509)	2/9036 (0.0002213)	0
c.2633G > A/p.Arg878His	1/582	8/277878 (0.00002879)	2/106914 (0.00001871)	3/19834 (0.0001513)	1/8972 (0.0001115)	0
c.2954C > T/p.Ser985Leu	1/582	-/-	-/-	-/-	-/-	-/-
c.3371C > T/p.Thr1124Met	1/582	2/251410 (0.000007955)	1/109394 (0.000009141)	0/18390 (0)	0/9044 (0)	0
c.10948A > G/p.Lys3650Glu	1/582	55/282826 (0.0001945)	18/120280 (0.0001497)	41/19948 (0.002055)	15/9962 (0.001506)	0
c.13396G > T/p.Val4466Leu	1/582	-/-	-/-	-/-	-/-	-/-
c.13924C > T/p.Leu4642Phe	2/582	19/282816 (0.00006718)	9/109408 (0.00008226)	19/19954 (0.0009522)	9/9064 (0.0009949)	0
c.14573A > G/p.Tyr4858Cys	1/582	10/250470 (0.00003992)	4/109132 (0.00003665)	10/18324 (0.0005457)	4/9022 (0.0004434)	0
c.3904 + 4 T > C/-	1/582	89/277064 (0.0003212)	34/118528 (0.0002869)	73/19308 (0.003781)	30/9950 (0.003015)	0
Total	10/582	189/250470	71/106914	151/18324	61/9036	
*P* value*		< 0.001	< 0.001	0.034	0.01	
OR (95% CI)		23.15(12.19–43.96)	26.31(13.50–51.26)	2.10(1.10–4.01)	2.57(1.31–5.05)	

*Abbreviations***:** *CI* Confidence interval, *gnomAD* Genome Aggregation Database, *OR* Odds ratio

^*^P-values and odds ratio were estimated with a 2-sided Fisher’s exact test

All *MDN1* variants identified were predicted to be damaging by more than one in silico tool (Supplementary Table S1). None of the five patients carried pathogenic or likely pathogenic variants in genes previously implicated in epilepsy [4].

### Clinical features

The five cases with *MDN1* variants had childhood-onset seizures with onset ages ranging from 1 to 7 years. The detailed clinical features are summarized in Table 1.

Three patients (Cases 1–3) were diagnosed with FS or FS + . The patient of case 1 had FS only once in early childhood. The other two patients (Cases 2 and 3) experienced infrequent FS/FS + and GTCS, and achieved seizure-free after treatment with valproate monotherapy. Interictal EEG for Case 2 revealed spike waves and spike-slow waves in the right parieto-occipital region, while Case 3 exhibited generalized sharp-slow waves. Brain MRI scans for both Cases 2 and 3 revealed no abnormalities. All three patients demonstrated normal neurodevelopment.

The remaining two patients (Cases 4 and 5) exhibited refractory seizures, which occurred five and seven years after brain damage, respectively. For Case 4, the patient experienced ABO hemolysis at birth. At the age of 7, the patient had frequent secondary GTCS or simple partial seizures (4–5 times/day). Brain MRI scans revealed bilateral parietal cortex atrophy and encephalomalacia (Fig. 2). For the Case 5, the patient suffered from subarachnoid hemorrhage and infection at 9 months of age. At the age of 6, the patient presented frequent complex partial seizures (3–4 times/day). Brain MRI scans detected atrophy of the left hemisphere. The interictal EEGs of both patients showed multifocal discharges. Both cases exhibited developmental disorders.

### Molecular alteration

**Fig. 3:**
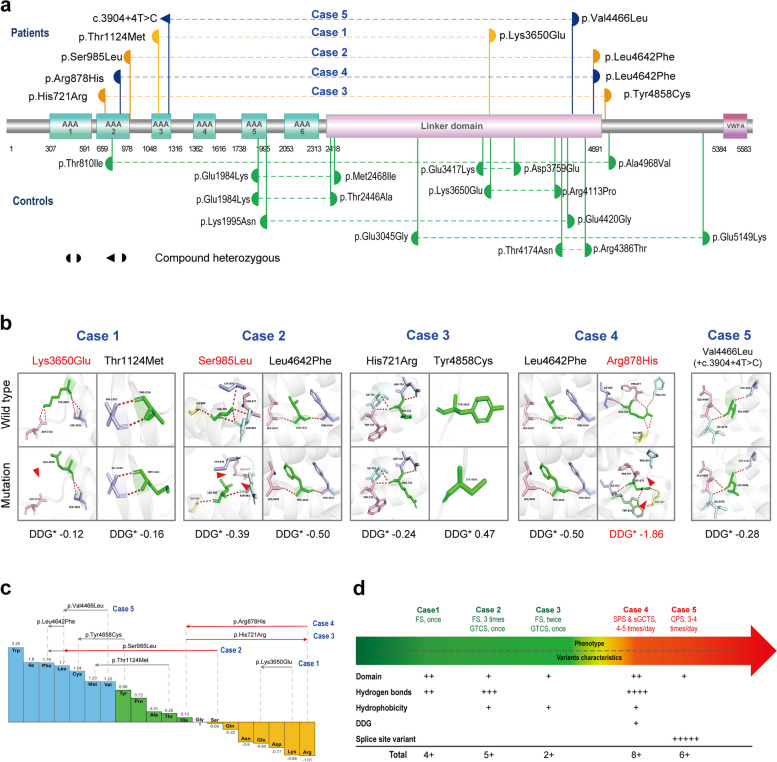
Schematic illustration of variant locations, hydrogen bond changes, and hydrophobicity alterations. **a** Linear schematic of missense *MDN1* variants and their locations on the *MDN1* protein. Variants identified in this study are shown above; variants identified in the control cohort are shown below. **b** Changes of hydrogen bonds and free energy change value (*DDG, kCal/mol) of the missense variants. The red dotted line represents hydrogen bonds. The red arrow indicates the change in hydrogen bonds. **c** Fauchère and Pliska hydrophobicity scale illustrates the hydrophobicity of 20 amino acids. Abscissa: from left to right, hydrophobicity gradually decreases. Blue amino acids are hydrophobic, green amino acids are neutral, and yellow amino acids are hydrophilic. Amino acids with high positive values are more hydrophobic, whereas amino acids with low negative values are more hydrophilic. **d** The variant characteristics of each case. Each alteration was assigned 1 point; variants in splice sites that may affect protein length were assigned 5 points. One " + " represents 1 point. Abbreviations: CPS, Complex partial seizure; FS, Febrile seizure; FS + , Febrile seizures plus; GTCS, Generalized tonic–clonic seizure; sGTCS, secondarily generalized tonic–clonic seizure; SPS, Simple partial seizure

The *MDN1* gene encodes a large protein consisting of 5696 amino acids, which includes six AAA domains and one MIDAS domain, connected by a lengthy linker domain. The specific locations of the variants in this study are presented in Fig. 3a**.** Among the biallelic variants identified in the cases, each pair was constituted by one variant located in the AAA2-AAA3 domains and the other located in the linker domain or its vicinity. In contrast, most of the variants from the asymptomatic control group were located outside the AAA domains.

Protein modeling, energy changes of single-point mutations, and hydrophobicity alterations were employed to assess the damaging effects of the variants. The results showed that three missense variants (p.Lys3650Glu, p.Ser985Leu, and p.Arg878His) were predicted to change hydrogen bonds with adjacent residues. Specifically, p.Lys3650Glu changed two hydrogen bonds, p.Ser985Leu changed three hydrogen bonds, and p.Arg878His changed four hydrogen bonds. The variant (p.Arg878His) was predicted to decrease protein stability, as indicated by DDG values of −1.86 kcal/mol (Fig. 3b). Additionally, three missense variants (p.Ser985Leu, p.His721Arg, and p.Arg878His) caused alterations in hydrophobicity (Fig. 3c). The biallelic variants in Case 1, who exhibited FS only once, did not cause hydrophobicity alteration; while the compound heterozygous variants in Cases 2–4, who manifested FS + or secondary epilepsy, had one of the paired variants with hydrophobicity alteration.

The damaging effects of each case were summarized in Fig. 3d, including the molecular subregional effects of the variants, hydrogen bond changes, hydrophobicity alterations, DDG changes, and variant types. Each alteration was assigned 1 point; while variants in splice sites that may affect protein length were assigned 5 points. Cases 1 to 3 exhibited relatively low scores, correlating with the presence of FS. In contrast, Cases 4 and 5 had relatively higher scores. These findings suggested a potential genotype–phenotype relationship, which may help explain the phenotypic heterogeneity observed among the different cases.

### Spatiotemporal expression profile of *MDN1*

**Fig. 4:**
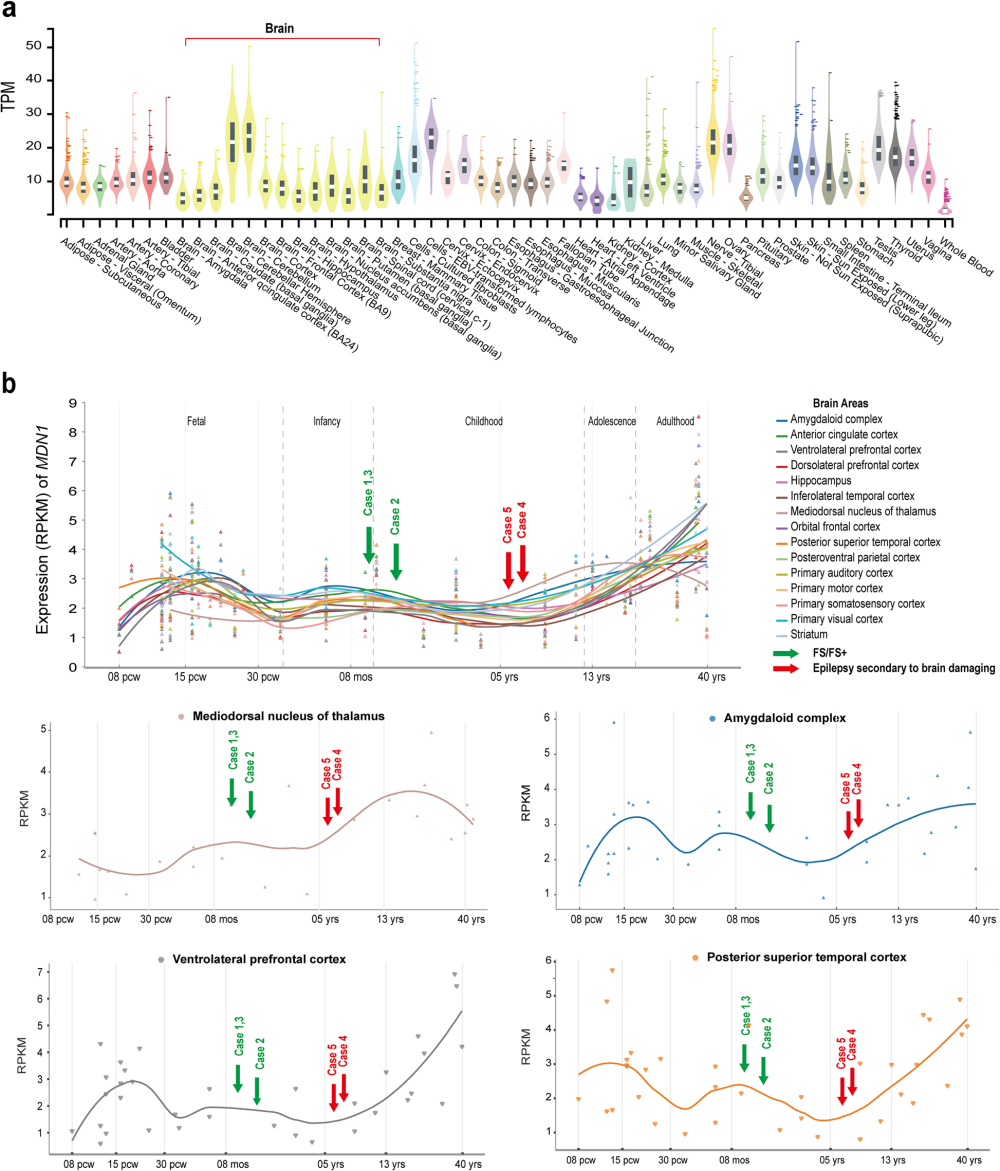
Genetic-dependent (expression) stage of *MDN1*. **a** RNA expression of *MDN1* in normal adult tissues. **b** Temporal expression of *MDN1* in various brain regions. Curves represent the temporal expression patterns of *MDN1* in different brain areas. The expression levels were retrieved from the human RNA-seq data in the BrainSpan database. The curve was fitted via the locally weighted scatterplot smoothing (LOWESS) method. Abbreviations: RPKM, Reads per kilobase per million mapped reads

Recent studies have indicated that the genetic-dependent (expression) stage is associated with the onset age of diseases. Building upon this understanding, we then investigated the spatiotemporal expression of *MDN1.* The gene is ubiquitously expressed in adult tissues, including multiple brain regions (Fig. 4a). *MDN1* expression occurs throughout the lifespan, with the first peak during the fetal period, followed by a slightly decrease during the perinatal period. The second expression peak appears in infancy, followed by a gradually increasing trend after the age of 5 and reaching the peak in adulthood (Fig. 4b). As reported previously [34], patients with FS/FS + have seizures in the infant stage, which is consistent with the second expression peak; while those with secondary epilepsy usually experience seizure onset at 6 to 7 years old, which is consistent with the initial stage of the third expression peak.

## Discussion

The Midasin AAA ATPase 1 (*MDN1*) gene (OMIM*618200), a member of the AAA protein family, plays a crucial role in ribosome maturation. Previously, the gene-disease association of *MDN1* remains undetermined. Compound heterozygous *MDN1* variants including eight missenses and one splicing variant were identified in five unrelated individuals with FS or secondary epilepsy. These variants were either absent or exhibit low frequencies in control groups and presented statistically higher frequencies in the case group compared to controls. All missense variants were predicted to be damaging by multiple in silico tool. The damaging effects were related to the specific molecular subregional effects and hydrophobicity alterations of *MDN1* variants. This study suggested that *MDN1* may be a susceptibility gene for epilepsy.

*MDN1* is predicted to have ATP binding activity and plays a crucial role in releasing or recycling various pre-60S ribosome assembly factors at different stages [8–10, 35]. Currently, the majority of research on the function of the *MDN1* gene is from the study of yeast. Orthologous *MDN1* proteins in humans and yeast have similar AAA ring structures, indicating that the protein structures are relatively conserved between the two species [36]. Previous studies have shown that the six AAA domains of *MDN1* form a cyclic hexamer. The MIDAS domain interacts with pre-60S ribosomal components by inserting into the AAA ring, mediating the release of multiple components from pre-60S ribosomes [8]. *Mdn1*’s C-terminal substrate-binding MIDAS domain can dock onto its N-terminal AAA ring. Conformational changes in the AAA domains can be directly transmitted to the MIDAS domain. The AAA2 H2 insertion may play an important regulatory role in the functionality of *MDN1*, as its conformational changes are closely related to docking with the MIDAS domain. The linker domain allows the MIDAS to dock onto the AAA ring, which is essential for ribosome biogenesis processes. Although *MDN1* is highly expressed in human brain throughout whole life, the association between *MDN1* and human disease remains unclear.

The pRec is a metric used to evaluate intolerance to biallelic loss-of-function (LOF) variants [37]. The *MDN1* gene has a pRec of 1.0 (> 0.9), indicating a high level of intolerance to biallelic LOF variants. The variants in *MDN1* were all compound heterozygous variants. These variants were identified in patients with FS or epilepsy secondary to brain damage, suggesting *MDN1* as a potential susceptibility gene for epilepsy. The frequencies of these variants were significantly higher in the case group compared to the controls, including both the frequency of compound heterozygous variants and the aggregate frequency of variants. Molecular subregional implication analysis showed the potential significance of the AAA2-AAA3 domains. These findings further support the gene-disease association.

In this study, the five patients exhibited phenotypic variation, including FS/FS + and refractory seizures secondary to brain damage. The biallelic variants in Case 1 did not change hydrophobicity, and the patient presented FS only once. The patients in Cases 2–4, who presented with FS + or secondary epilepsy, had one of paired variants with hydrophobicity alteration. Particularly, the patients in Cases 4 and 5, both with acquired brain damage, experienced refractory frequent seizures. These findings suggest that *MDN1* variants cause susceptibility to epilepsy; other factors, such as molecular alterations and acquired brain abnormalities, potentially play a role in determining the severity of clinical manifestations.

In this study, the first seizure occurred 5 years and 7 years after brain damage for Cases 4 and 5, respectively. Although the brain structural abnormalities caused by acquired etiologies may contribute to the development of epilepsy, seizures did not occur immediately following the brain damaged. Therefore, it is considered that genetic factors may have altered the epilepsy susceptibility in these patients. However, the interaction between genetic factors and acquired factors warrants further investigations. The spatiotemporal expression analysis showed that *MDN1* is expressed throughout the lifespan, with three expression peaks in the brain. Notably, its expression gradually increases after the age of five and reaches a peak during adulthood. The age of epilepsy onset in the two patients coincides with the initial stage of the third expression peak. Genes differ in the genetic dependent features, including the vital consequence of gene loss (genetic dependent nature), the low quantitative limit of genetic function required for normal life (genetic dependent quantity), and the temporal pattern of expression of genes (genetic dependent stage) [38], which are associated with the pathogenic potential of genes and the clinical characteristics of genetic diseases [21, 34, 37, 39–41]. Epilepsy is a complex clinical entity with phenotypes of varied severity, such as severe developmental and epileptic encephalopathy, mild idiopathic epilepsies with favorable outcomes, and susceptibility to epilepsy. Studies focusing on genetic-dependent features are necessary to explore the underlying mechanisms. Additionally, further research is required to determine whether *MDN1* variations have long-term effects on neural network remodeling and whether brain damage triggers the delayed manifestation of genetic susceptibility.

This study has several limitations. Firstly, it primarily relies on clinical and genetic evidence, and future experimental studies are needed to validate these findings. Additionally, the sample size is limited, highlighting the need for further studies with larger cohorts.

## Conclusions

In conclusion, this study suggests that *MDN1* variants contribute to the susceptibility to epilepsy. The molecular subregional implications and hydrophobicity alterations of *MDN1* potentially play a role in the pathogenesis of epilepsy.

## Web resources

AlphaFold protein structure database: https://alphafold.ebi.ac.uk/;

Brainspan database: http://www.brainspan.org;

Evo-devo mammalian organs database: http://www.apps.kaessmannlab.org/evodevoapp/;

GD&P Database: https://www.gdap.org.cn/;

I-Mutant 2.0 program: https://folding.biofold.org/cgi-bin/i-mutant2.0.cgi;

VarSite web: http:/www.ebi.ac.uk/thornton-srv/databases/VarSite.

## Supplementary Information

Supplementary Material 1

## Data Availability

The data supporting the findings of this study are available from the corresponding author upon reasonable request.

## Abbreviations

AAA: ATPase associated with various activities
ASMs: Anti-seizure medications
FS: Febrile seizures
FS +: Febrile seizure plus
GTCS: Generalized tonic–clonic seizure
GWAS: Genome-wide association studies
MAF: Minor allele frequency
MIDAS: Metal ion dependent adhesion site
MRI: Magnetic resonance imaging
SNPs: Single nucleotide polymorphisms
VEEG: Video electroencephalogram
