# Supplementary material for: MDN1 variants cause susceptibility to epilepsy: For the China Epilepsy Gene 1.0 Project
Source: Acta Epileptol. 2025 Mar 3;7:17. doi: 10.1186/s42494-025-00209-3 (PMC11960335; doi:10.1186/s42494-025-00209-3)
Supplement: Supplementary file 1 — Supplementary Material 1 [file 42494_2025_209_MOESM1_ESM.docx]

**Table S1** Genetic features of the individuals with *MDN1* variants

| **cDNA change** | **Protein change** | **Domain** | **MAF** | **DDG**  **(kcal/mol)** | **Mutation**  **Taster** | **CADD** | **FATHMM_MKL** | **fitCons** | **ReVe** | **GERP++** | **phyloP** | **phastCons** | **SiPhy** |
| --- | --- | --- | --- | --- | --- | --- | --- | --- | --- | --- | --- | --- | --- |
| c.2162A>G | His721Arg | AAA2 | 2.13*10^-5^ | -0.24 | DC(0.877) | T(13.41) | D(0.987) | D(0.732) | B(0.118) | C(3.4) | C(3.762) | C(1) | C(13.019) |
| c.2633G>A | Arg878His | AAA2 | 2.88*10^-5^ | -1.86 | DC(1) | D(25.5) | D(0.972) | D(0.707) | B(0.417) | C(4.09) | C(3.672) | C(1) | C(12.537) |
| c.2954C>T | Ser985Leu | - | - | -0.39 | DC(1) | D(33) | D(0.999) | D(0.707) | P(0.992) | C(5.54) | C(9.568) | C(1) | C(19.487) |
| c.3371C>T | Thr1124Met | AAA3 | 7.96*10^-6^ | -0.16 | DC(1) | D(28) | D(0.994) | D(0.707) | P(0.793) | C(5.55) | C(9.178) | C(1) | C(19.591) |
| c.10948A>G | Lys3650Glu | Linker | 1.95*10^-4^ | -0.12 | DC(0.991) | T(16.36) | D(0.823) | D(0.719) | B(0.037) | NC(-0.602) | NC(1.515) | NC(0.982) | NC(9.907) |
| c.13396G>T | Val4466Leu | Linker | - | -0.28 | PL(0.975) | T(8.484) | D(0.747) | D(0.707) | B(0.028) | NC(-1.1) | NC(-0.038) | NC(0.744) | NC(0.011) |
| c.13924C>T | Leu4642Phe | Linker | 6.72*10^-5^ | -0.50 | DC(1) | D(31) | D(0.988) | D(0.707) | P(0.626) | C(5.77) | C(4.775) | C(1) | NC(11.691) |
| c.14573A>G | Tyr4858Cys | - | 3.99*10^-5^ | 0.47 | DC(1) | D(27.3) | D(0.970) | D(0.707) | P(0.646) | C(5.26) | C(4.792) | C(1) | C(15.470) |
| c.3904+4T>C | - | - | 3.21*10^-4^ |  | - | - | - | - | - | - | - | - | - |

**Abbreviations:** B, Benign; C, Conserved; CADD, Combined annotation dependent depletion; D, Damaging; DC, Disease causing; Fathmm-MKL, Functional Analysis through Hidden Markov Models-Multiple Kernels Learning; fitCons, fitness conservation score; GERP++, Genomic Evolutionary Rate Profiling; MAF, Minor allele frequency from Genome; NC, Nonconserved; P, Pathogenic; phastCons, Phylogenetic Analysis with Space/Time models conservation scoring and identification of conserved elements; phyloP, Phylogenetic Analysis with Space/Time models Computation of p-values for conservation or acceleration, either lineage-specific or across all branches; T, Tolerable; PL, Polymorphism.
